# Testing for clustering at many ranges inflates family-wise error rate (FWE)

**DOI:** 10.1186/1476-072X-14-4

**Published:** 2015-01-15

**Authors:** Matthew Shane Loop, Leslie A McClure

**Affiliations:** Department of Biostatistics, University of Alabama at Birmingham, 1665 University Boulevard, RPHB 327, 35294 Birmingham, Alabama USA

**Keywords:** Ripley’s K function, Overall clustering, Point process, Family wise error rate (FWE), Multiple testing

## Abstract

**Background:**

Testing for clustering at multiple ranges within a single dataset is a common practice in spatial epidemiology. It is not documented whether this approach has an impact on the type 1 error rate.

**Methods:**

We estimated the family-wise error rate (FWE) for the difference in Ripley’s *K* functions test, when testing at an increasing number of ranges at an alpha-level of 0.05. Case and control locations were generated from a Cox process on a square area the size of the continental US (≈3,000,000 mi^2^). Two thousand Monte Carlo replicates were used to estimate the FWE with 95% confidence intervals when testing for clustering at one range, as well as 10, 50, and 100 equidistant ranges.

**Results:**

The estimated FWE and 95% confidence intervals when testing 10, 50, and 100 ranges were 0.22 (0.20 - 0.24), 0.34 (0.31 - 0.36), and 0.36 (0.34 - 0.38), respectively.

**Conclusions:**

Testing for clustering at multiple ranges within a single dataset inflated the FWE above the nominal level of 0.05. Investigators should construct simultaneous critical envelopes (available in spatstat package in R), or use a test statistic that integrates the test statistics from each range, as suggested by the creators of the difference in Ripley’s *K* functions test.

## Background

When studying chronic disease in large cohort studies, where geographic location of participants’ residences are known, we can investigate whether disease rates, or risk factors for the disease, vary geographically over the region of interest (e.g., REasons for Geographic and Racial Differences in Stroke [REGARDS] study [1]). Geographic variation in these factors can result from variation in demographics, environmental exposures, dietary patterns, or healthcare quality, among other things. When we observe spatial variation in a chronic disease, a common next step is to determine whether there is spatial variation in the most important risk factors.

### Difference in Ripley’s *K*functions test

The most popular test of overall clustering among epidemiologists when locations of cases and controls are known is the difference in Ripley’s *K* functions test [2–4]. Epidemiologists appreciate this method because it can be used with inhomogeneous distributions of cases and controls, a type of spatial process where the mean number of points per unit area varies across the region of interest. Population density varies across the US, being highest in cities, so residential locations often follow an inhomogeneous spatial distribution. The difference in *K* functions test determines whether there is clustering in the cases *above and beyond* the clustering observed in the controls. The general form of this test is, given an *a priori* range *h*,


where *K*_*i*_(*h*)=*λ*^−1^*E* [ *#* of events of type i within h of a randomly chosen event of type *i*], *i*∈ (case, control) and *λ* is the expected number of points per unit area (“intensity”). The significance of an observed value, , is often determined by Monte Carlo procedures, where the labels of “case” or “control” are randomly permuted among the observed event locations. These random permutations produce a distribution of  under the null hypothesis of no clustering, to which the observed value of  can be compared.

Because of the exploratory nature of the test, investigators often set many values for *h*, and evaluate the significance of , where *i*=1,…,*r*. Many ranges are tested because the observed dataset could have been generated by a spatial process at any of several geographic scales. The original work describing the difference in Ripley’s *K* functions test provided an overall test for clustering, which incorporated all of the values for  into one test statistic (Equation 8 in [3]). Few studies that cite the difference in *K* functions statistic by Diggle and Chetwynd (1991) use this overall test statistic.Instead, they evaluate  at each range individually. For an example of a paper that did use the integrated test statistic, see [5]. Most investigators choose *r* values for *h*, and then effectively perform *r* hypothesis tests and conclude that there is evidence for clustering up to ranges *h*_*i*_ [6], or that there is evidence of clustering in the overall dataset [7]. The potential for type 1 error inflation in this context has been discussed in the the ecology literature [8], but little attention is given to the magnitude of potential inflation of the type 1 error rate, especially as a function of the number of rangestested.

### Simulation study of FWE when testing at multiple ranges

We estimated the family-wise error rate (FWE), or the probability of committing at least one type 1 error (i.e., a false conclusion that clustering exists when it does not), when testing for clustering at increasing numbers of ranges. It is not currently known if, and if so to what extent, the FWE becomes inflated above the nominal level when using the difference in *K* functions test at multiple ranges within a single dataset. We report the results of a simulation study using datasets the size of the REGARDS cohort (approximately 30,000 participants), with discussion.

## Results and discussion

**Figure 1:**
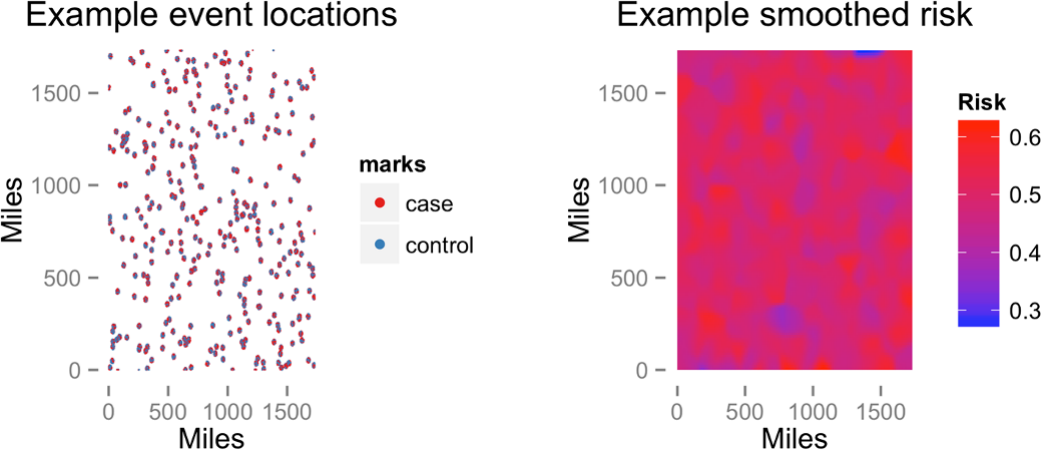
**Example simulated dataset.** Point map of locations of cases and controls, each simulated from independent Cox processes with expected sample sizes of 15,000 events, as well as smoothed risk estimates using Nadaraya-Watson estimator.

Figure 1 shows a plot of the locations and case status of an example dataset from the simulation, as well as a map of smoothed risk for being a case. Clusters of points were generated, with the radius of the clusters relatively small compared to the total area, in order to mimic human population distribution. The numbers of cases and controls within each cluster were approximately equal. The mean(±sd) sample size was 29,980(±1,746), and the mean(±sd) proportion of cases was 0.50(±0.002).

### Estimated FWE

The estimated type 1 error rates and 95% confidence intervals are presented in Table 1. The FWE was conserved at the nominal level of 0.05 when only one range was tested [FWE (95% confidence interval): 0.05(0.04 - 0.06)]. The FWE increased in a nonlinear fashion as the number of ranges tested increased. The FWE for 10 tests was 0.22 (0.20 - 0.24), for 50 tests was 0.34 (0.31 - 0.36), up to a maximum of 0.36 (0.34 - 0.38) when 100 ranges were tested.

**Table 1:** **Estimated FWE and 95% confidence intervals, by number of ranges tested**

Number of tests	FWE (95% confidence interval)
1	0.05 (0.04 - 0.06)
10	0.22 (0.20 - 0.24)
50	0.34 (0.31 - 0.36)
100	0.36 (0.34 - 0.38)

### Discussion

Our simulation study has shown that when testing for overall clustering using a large cohort that covers a large area, we should use caution when testing for clustering at many ranges. Multiple testing is well-known to endanger proper inference by inflating the probability of a false positive. Although the potential for inflation of the FWE is clear when testing for clustering at multiple ranges, the magnitude of the inflation has not been documented. Specifically, if *r* null hypotheses of constant risk are tested, then the probability of falsely finding evidence of clustering, when none exists at any ranges, is quite high. For instance, testing 10 ranges is not uncommon, and doing so can lead to a FWE of 0.2, which is four times the usual nominal rate of 0.05. In large cohort studies like REGARDS, however, there are far more than 10 potential ranges one could test, which could lead to even further inflation of the FWE.

Beyond the danger of wrong conclusions for the difference in *K* functions test is the danger that an error could be carried through to downstream analyses. For instance, an investigator might want to use a Bayesian hierarchical model for creating a map of risk for the disease, and might use the ranges suggested by the difference in *K* functions test as informative priors for the range parameter of a covariance function. Or, an investigator using semi parametric regression for smoothing might choose the number of basis functions based upon the number of ranges suggested by the difference in *K* functions test. These errors could lead to insufficient or excessive allocation of public health resources to particular areas.

#### Correlation among the tests

**Figure 2:**
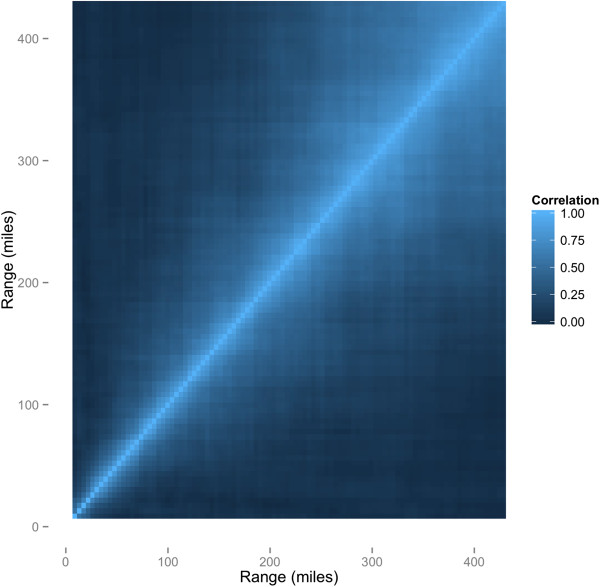
**Correlations among tests when testing 100 ranges.** Estimated correlations of the outcome of whether the null hypothesis was rejected or not among the 100 ranges tested.

Although the FWE is inflated when testing multiple ranges, the inflation is not as severe as if the same number of independent statistical tests were performed, due to the correlation among tests at nearby ranges. Figure 2 further illustrates this point, showing the high correlation between tests at neighboring ranges. Readers of studies testing for clustering at multiple ranges using the difference in Ripley’s *K* functions test should acknowledge this decreased inflation of the FWE, compared to studies with independent tests, in order to draw proper conclusions. We note that, keeping the number of tests constant, the FWE could be decreased by decreasing the maximum range tested, and/or the interval between the tests. The decrease in FWE would be caused by the increased correlation among the tests, thus decreasing the effective number of independent tests. However, investigators often want to test for clustering at as many ranges as is feasible, in order to not miss evidence of clustering at a particular scale. Decreasing the maximum range tested and/or the interval between the tests might not be an optimal strategy.

#### Recommendations

Two approaches can keep the FWE near the nominal level when testing multiple ranges. The first is to use a test statistic which integrates the values of  over all values of *h*, taking into account the number of ranges tested, and provides the most suggestive scale of spatial clustering [9]. This procedure achieves the goal of combining information from all ranges tested, similar to the statistic provided in Equation 8 of Diggle and Chetwynd (1991), while having the added benefit of suggesting a particular scale for the clustering. One such method was originally suggested by Diggle and Chetwynd (1991) in Equation 9. This method defines the statistic


which has the approximate distribution under the null hypothesis given in Equation 10 of their manuscript, as long as *r* is large enough. This method can be implemented in the splancs package of the R statistical environment by using the khvc function to calculate the variances of estimates  at different ranges [10, 11]. A limitation of using this method is that splancs has no option to change the edge correction used. Thus, for larger epidemiological cohorts like REGARDS, the border edge correction cannot be used, and computation time might be prohibitive. This lack of ability to change the edge correction, as well as the additional steps needed from the user to compute the overall statistic, might deter investigators from using this method.

Other methods that account for testing multiple ranges while suggesting a specific scale of clustering are Tango’s MEET [12] and some of the likelihood based methods proposed in [13]. These methods identify the scales most suggestive of clustering, and then use the *p*-value at that scale as the test statistic [12, 13]. The magnitude of the FWE inflation shown in our study suggests increased use of the alternative methods described here, as opposed to testing multiple ranges individual using the difference in Ripley’s *K* functions test.

A second approach to temper FWE inflation is to use simultaneous critical regions when evaluating the significance of the difference in *K* functions. When using simultaneous critical envelopes, the null hypothesis of constant risk is rejected if any of the  lie outside the critical envelopes. Further details of the envelope function in the spatstat package can be found in the documentation [14] in R.

Both of the two methods mentioned here essentially reduce *r* hypothesis tests to one hypothesis test. The one hypothesis test is whether there is evidence of clustering at any range *h*, and if so, suggests the most likely range. A final approach might be to adjust the critical envelopes to correct for the multiple testing, while accounting for the correlation structure among the tests. For instance, the estimated covariances between the  at different ranges could be used to derive the covariances among the tests themselves. However, the overall test statistic and simultaneous critical envelopes are already implemented in common software, and can serve to test for clustering at *any range*.

#### Study strengths

This study simulated conditions that could reasonably occur in public health settings, which usually have a few thousand observations [15–17]. Additionally, with the move towards “big data” by government organizations [18] and industry, both the number of observations and the size of the areas being routinely studied could increase substantially. Increased sample sizes and study areas bring increasingly larger numbers of potential ranges to test for evidence of clustering. Therefore, inflation of FWE might become a concern in public health studies with spatially-referenced data.

Both the case and control locations in the simulated datasets were generated using an inhomogeneous process. Thus, the results of this study can be applied to many datasets documenting human disease patterns where point locations of cases and controls are available.

Finally, this study has been conducted in a reproducible manner, with all code available at github.com/mloop/kdiff-type1-error-rate. Other investigators can modify the code and investigate how the FWE could be affected in their own contexts.

#### Limitations

The results of this simulation study can be directly applied only to datasets with tens of thousands of observations, spanning an area the size of the continental US. We suspect that the number of effectively independent tests would increase with an increase in the intensity (number of points per unit area), an increase in the area of the region of interest, or both. An increase in the number of independent tests could inflate the FWE to a greater degree than we observed in this study.

A minor limitation was that the lengths of the confidence intervals for the FWEs across the different number of ranges tested are not of identical length. This limitation occurred for two reasons. First, the number of simulations performed was chosen to produce confidence intervals of length 0.02 assuming the nominal FWE, but the width of the confidence interval for the mean of a Bernoulli distribution, *p*, depends upon the value of *p*. Since the FWEs were not known before the simulation study, but were estimated, we had no way to know how many iterations each condition would need to produce confidence intervals of identical lengths. Second, eight nodes of the High Performance Computing cluster failed during the computation, producing marginally different sample sizes for the estimates of the FWE. However, the differing lengths of the confidence intervals do not change our interpretation of the results.

## Conclusions

Testing multiple ranges for evidence of clustering using the difference in *K* functions appreciably inflates the FWE. Investigators should use a statistic that combines the estimates of *D*(*h*_*i*_) at all ranges, use simultaneous critical envelopes, or modify the critical envelopes to account for multiple correlated tests when testing multiple ranges for evidence of clustering.

## Methods

### Generation of datasets

The datasets were generated from a Cox process [19], using the spatstat v. 1.31-2 package [14] in the R v. 3.0.1 statistical environment [10]. For each dataset, a set of “parent” events were generated from a homogeneous Poisson point process, with intensity *κ*=0.0001, onto a square region the size of the continental US (3,000,000 mi ^2^). Then, a random number of “child” events were generated from a Poisson distribution with mean *λ*=100, within a circle of radius 15 mi. For each parent point, both case and control children were generated independently. For each of the four sets of ranges tested (1, 10, 50, or 100 ranges), we generated 2,000 datasets, leading to a total of 8,000 datasets. We chose 2,000 datasets for estimation of the FWE for each set of ranges in order to achieve a 95% confidence interval length of 0.02 for the estimated FWE, assuming the nominal significance level of 0.05. Each dataset was generated using a different random seed, with seeds 30,000 numbers apart. The Mersenne-Twister random number generator was used [20]. The code that generated the datasets can be found at github.com/mloop/kdiff-type1-error-rate/blob/master/data/genesis.R.

### Estimating FWE

Using the spatstat v. 1.31-2 package in R v. 3.0.1, for each range tested in each dataset, we estimated the value for the difference in *K* functions, . The ranges were chosen by dividing the interval from 0 to one quarter the length of a side of the simulated region into a number of equidistant intervals equal to the number of tests being done. The code to calculate the difference in *K* functions was taken from [21]. The border edge correction was used for all estimates of the *K* function, given the expected sample sizes of the datasets (15,000 cases and 15,000 controls) [22]. Then, we performed 199 random labelings to generate the null distribution of values for  at each range. Although some investigators use larger numbers of Monte Carlo replicates, it has been shown by previous investigators that 39 Monte Carlo replicates can be sufficient to create Monte Carlo tests with power approximately equal to the power of tests based upon true sampling distributions, such as the Gaussian, *t*, and exponential distributions [23]. Based upon this work, Diggle (2014) suggests that 99 Monte Carlo replicates is sufficient for a one-sided test (p. 14 of [24]). Therefore, we approximately doubled the number of Monte Carlo replicates for the two-sided test using the difference in Ripley’s *K* functions. The choice of 199 Monte Carlo replicates balanced computation time for the simulation against sufficient numbers of Monte Carlo replicates that provide power approximately equal to a test based upon the true sampling distribution of  under the null hypothesis, which is unknown. Random labeling refers to the “random” permutation of the labels “case” and “control” among the observations in a dataset, keeping the total number of cases and controls constant. Random labeling generates data under the null hypothesis of constant risk [25]. The 5th- and 195th-ranked simulated values of  were used as critical values, providing a two-sided size *α*=0.05 test at each range. Again, the Mersenne-Twister random number generator was used, along with seeds that were 30,000 numbers apart between iterations.

A rejection of the null hypothesis for a single iteration was defined as an observed value for  being outside the critical regions *for any value of h*_*i*_, *i*=1,…,*r*. We estimated the FWE for each condition (1, 10, 50, or 100 ranges tested) by dividing the number of rejections by the number of iterations (2,000; 1,998; 1,997; and 1,997, respectively). The number of iterations were just under 2,000 for the 10, 50, and 100 range conditions due to the failing of 8 nodes on the HPC cluster during specific runs of the simulation. We chose to regard this number of missing iterations as trivial, and unlikely to affect our conclusions. Approximately Gaussian 95% confidence intervals were estimated for each estimated FWE. The code that calculated the values for  can be found at https://github.com/mloop/kdiff-type1-error-rate/blob/master/analysis/simulation.R , and the code that estimated the values for  can be found at https://github.com/mloop/kdiff-type1-error-rate/blob/master/analysis/summarize.Rmd .

### Hardware and software

Dataset generation and type 1 error rate estimation was performed on the University of Alabama at Birmingham’s (UAB) high performance computing (HPC) cluster. Plots were created using the ggplot2 package in R [26]. Data manipulation was performed using the dplyr and plyr packages in R [27, 28]. Plot creation and data manipulation were done on a 2012 Mac Mini.
